# Clinicians' adherence versus non adherence to practice guidelines in the management of patients with sarcoma: a cost-effectiveness assessment in two European regions

**DOI:** 10.1186/1472-6963-12-82

**Published:** 2012-03-28

**Authors:** Lionel Perrier, Alessandra Buja, Giuseppe Mastrangelo, Antonella Vecchiato, Paolo Sandonà, Françoise Ducimetière, Jean-Yves Blay, François Noël Gilly, Carole Siani, Pierre Biron, Dominique Ranchère-Vince, Anne-Valérie Decouvelaere, Philippe Thiesse, Christophe Bergeron, Angelo Paolo Dei Tos, Jean-Michel Coindre, Carlo Riccardo Rossi, Isabelle Ray-Coquard

**Affiliations:** 1 University of Lyon, F-69007 Lyon; CNRS, GATE Lyon-St Etienne, UMR n°5824, 69130 Ecully, Department Cancer and Environment, Cancer Centre Léon Bérard, 69008 Lyon, France; 2 Department of Environmental Medicine and Public Health, University of Padova, 35122 Padova, Italy; 3 Department of Environmental Medicine and Public Health, Padua University, 35122, Padova, Italy; 4 Melanoma and Sarcomas Unit, Veneto Institute of Oncology, IOV, IRCCS, 35128, Padova, Italy; 5 Department of Environmental Medicine and Public Health, Padua University, 35122 Padova, Italy; 6 University of Lyon, Cancer Centre Léon Bérard; Santé-Individu-Société EA-INSERM 4129, 28 rue Laënnec, 69373 Lyon, Cedex 08, France; 7 University of Lyon, Department of Medical Oncology, Cancer Centre Léon Bérard, 28 rue Laennec, 69373 Lyon, Cedex 08, France; 8 Department of Digestive Surgery, University Hospital Lyon Sud, 165 Chemin du Grand Revoyet, 69310 Pierre Bénite, France; 9 ERIC EA 3083, University of Lyon (University Claude Bernard Lyon 1), 69622 Lyon, France; 10 University of Lyon, Cancer Centre Léon Bérard, Department of Medical Oncology, 28 rue Laënnec, 69373 Lyon, Cedex 08, France; 11 Department of Anatomopathology, Centre Léon Bérard, 69373 Lyon, Cedex 08, France; 12 Department of Anatomopathology, Centre Léon Bérard, 69373, Lyon, Cedex 08, France; 13 Department of Imaging, Centre Léon Bérard, 69373 Lyon, Cedex 08, France; 14 Institut d'Hémato-Oncologie Pédiatrique, 1, place Professeur Joseph Renaut, 69008 Lyon, France; 15 Department of Pathology, Hospital of Treviso, Piazza Ospedale 1, 31100 Treviso, Italy; 16 Department of Pathology, Institut Bergonie, 229 Cours De l'Argonne, 33076 Bordeaux, France; 17 Melanoma and Sarcomas Unit, Veneto Institute of Oncology, IOV, IRCCS, University of Padova, 35128 Padova, Italy; 18 University de Lyon, Centre Léon Bérard, Department of Medical Oncology, 28 rue Laennec, 69008 Lyon, INSERM EA 4129 « SIS », 28 rue Laennec, 69008 Lyon, France

**Keywords:** Sarcoma, Cancer, Clinical practice guidelines, Adherence, Compliance, Cost-effectiveness

## Abstract

**Background:**

Although the management of sarcoma is improving, non adherence to clinical practice guidelines (CPGs) remains high, mainly because of the low incidence of the disease and the variety of histological subtypes. Since little is known about the health economics of sarcoma, we undertook a cost-effectiveness analysis (within the CONnective TIssue CAncer NETwork, CONTICANET) comparing costs and outcomes when clinicians adhered to CPGs and when they did not.

**Methods:**

Patients studied had a histological diagnosis of sarcoma, were older than 15 years, and had been treated in the Rhône-Alpes region of France (in 2005/2006) or in the Veneto region of Italy (in 2007). Data collected retrospectively for the three years after diagnosis were used to determine relapse free survival and health costs (adopting the hospital's perspective and a microcosting approach). All costs were expressed in euros (€) at their 2009 value. A 4% annual discount rate was applied to both costs and effects. The incremental cost-effectiveness ratio (ICER) was expressed as cost per relapse-free year gained when management was compliant with CPGs compared with when it was not. To capture uncertainty surrounding ICER, a probabilistic sensitivity analysis was performed based on a non-parametric bootstrap method.

**Results:**

A total of 219 patients were included in the study. Compliance with CPGs was observed for 118 patients (54%). Average total costs reached 23,571 euros when treatment was in accordance with CPGs and 27,313 euros when it was not. In relation to relapse-free survival, compliance with CPGs strictly dominates non compliance, i.e. it is both less costly and more effective. Taking uncertainty into account, the probability that compliance with CPGs still strictly dominates was 75%.

**Conclusions:**

Our findings should encourage physicians to increase their compliance with CPGs and healthcare administrators to invest in the implementation of CPGs in the management of sarcoma.

## Background

Sarcomas are rare tumours (accounting for only 1-2% of all cancers) originating from connective tissue, skin, retroperitoneum, bone and viscera [1]. The rarity of the disease, along with the variety of histological types and locations and the heterogeneity of prognostic factors associated with local or distant spread, mean that physicians have only limited personal experience of managing the disease. Furthermore, outside centres of excellence, there is little graduate or post-graduate medical training in its optimum management. To improve the diagnosis and prognosis of sarcoma, the European Commission funded the Connective Tissue Cancer Network (CONTICANET) aimed at increasing the standardization of diagnostic and therapeutic procedures.

In order to reduce inappropriate medical procedures, Clinical Practice Guidelines (CPGs) were developed by the Fédération Nationale des Centres de Lutte contre le Cancer (the French Federation of Comprehensive Cancer Centres) [2] and by the Italian National Research Council [3]. France and Italy reached a consensus in their CPGs relating to all phases of sarcoma management (initial examination and diagnosis, histopathological report, surgery, chemotherapy, and radiation therapy) except surveillance after therapy (see Annexe 1).

The impact of adherence to CPGs has received some research attention [4-10], even in the management of rare cancers [11-14]. However, little is known about the financial impact of clinicians' adherence to CPGs in general, and the impact of adherence on outcomes and costs of care has only rarely been simultaneously considered [15]. Such assessments are of particular value in the current period of budgetary constraint, which prevents the achievement of improved cancer outcomes through increased health expenditure [16,17].

We therefore assessed the cost-effectiveness of compliance with CPGs in sarcoma management by investigating the relationship between health outcome and resource consumption in patients treated in the regions of the Rhône-Alpes in France and Veneto in Italy.

## Methods

### Study design

Our starting point was 327 sarcoma patients aged ≥ 15 years (254 in Veneto and 73 in Rhône-Alpes) diagnosed over the relevant periods in the two regions. Absence of patient consent, care undertaken outside the participating regions or in private hospitals, and missing records (Table 1) reduced the number of patients included in the study to 219, 58 from Rhône-Alpes and 161 from Veneto. These patients were followed retrospectively for the three years after sarcoma diagnosis or until the date of death. All patients had histological confirmation of primary malignant sarcoma, with or without distant metastasis at initial diagnosis. With the exception of osteosarcoma, sarcomatoid carcinoma, mesothelioma, neuroblastoma, paraganglioma and mixed (epithelial and mesenchymal) tumours of the female genital tract, all histological subtypes were included. All patients in Rhône-Alpes had been diagnosed between March 2005 and February 2006 and treated at the University Hospital of Lyon and/or at the Léon Bérard Cancer Centre. All patients in Veneto had been diagnosed between January 2007 and December 2007 and treated in the public hospitals of the region. Patients were managed in accordance with the ethical principles for medical research involving human subjects described in the Declaration of Helsinki. The study received approval in France from the National Ethics Committee (N°904073) and the National Committee for Protection of Personal Data (N°05-1102), and from the Local Sanitary Agency of the Veneto Region and the Ethics Committee of the Azienda Ospedaliera di Padova (N°156/06/CE) in Italy. Each patient was required to give signed informed consent. Lack of informed consent and treatment of sarcoma outside the Veneto or Rhône-Alpes were exclusion criteria. Cases of relapsed disease were also excluded since CPGs for this setting are not available.

**Table 1:** Attrition of the study population

Region	Eligible	No consent	Care outside the region	Data not available^@^	Included
Veneto	254	55	17	21	161
Rhône-Alpes	73	0	6	9	58
Total	327	55	23	30	219

^@^patients treated at private hospitals; missing clinical records

### Clinical data

Hospital records were used to obtain data on the characteristics of patients (age, sex, comorbidities) and their sarcomas (visceral or soft tissue; superficial or deep tumour); localization in lower or upper limb, head-neck or trunk; histological subtype; major tumour diameter at imaging and surgery; and grade). Data were also obtained on resources utilization (using a micro-costing approach) at diagnosis and during surgery (primary and wide surgical resection), chemotherapy (drugs administered in hospital or outpatient facilities) and radiotherapy (sessions in hospital or outpatient facilities). We also obtained data covering relapse or metastasis during follow up, along with any subsequent need for surgical interventions, chemotherapy and/or radiotherapy. Information was collected on number of days of hospitalization, use of pathology resources such as micro-biopsy and cytology), use of imaging, and supportive treatments such as antibiotics. The date of relapse was used to calculate relapse free survival from diagnosis.

Physicians independent of the study (two from the Léon Bérard Cancer Centre and two from the University of Padua) assessed whether or not there had been compliance with CPGs in each management phase covered by such guidelines. Overall management was considered to have been compliant only when CPGs had been followed at all stages of diagnosis, treatment, and follow-up.

### Costs and indicators of effectiveness

Costs were assessed for each patient and from the hospital's point of view for the period between diagnosis and the end of follow-up or death [18]. Sources of unit costs and prices are described in Table 2[19-21]. Days of hospital admission were multiplied by the cost per day (taking the average of the 2009 costs in France and Italy). This covered the cost of personnel, medications (except for chemotherapy and blood transfusions), use of medical devices, laboratory tests, depreciation of equipment and overheads. Doses of chemotherapy, and number of transfusions, radiotherapy sessions, imaging procedures, biopsies and consultations were multiplied by their respective unit costs (again taking the average of 2009 prices in France and Italy). Discounting of 4% per year was applied. The mean costs were calculated for patients whose overall management had been compliant with CPGs and for patients in whom it had not.

**Table 2:** Main unit costs and prices

Items		Unit costs and prices	Sources of information
Hospitalization (per day)		760,14€	Hospital Managers
Biopsy		51,45 €	[19,20]
Consultation (external)		68,73€	[19,20]
Radiotherapy (per session)		94,21€	[19,20]
Chemo-therapy(per milligram of drugs)	Caelix	17,74€	Hospital pharmacists
	Carboplatin	0,21€	Hospital pharmacists
	Cisplatin	0,32€	Hospital pharmacists
	Deticene	0,03€	Hospital pharmacists
	Doxorubicin	0,71€	Hospital pharmacists
	Etopophos etoposide	0,16€	Hospital pharmacists
	Gemcitabine	0,17€	Hospital pharmacists
	Holoxan	0,07€	Hospital pharmacists
	Ifosfamide	0,10€	Hospital pharmacists
	Imatinib	0,24€	Hospital pharmacists
	Melphalan	0,01€	Hospital pharmacists
	Navelbine	1,91€	Hospital pharmacists
	Oxaliplatin	3,92€	Hospital pharmacists
	Paclitaxel	2,25€	Hospital pharmacists
	Vincristine	7,16€	Hospital pharmacists
Transfusion (per pack)	Ps	121,33€	[19,21]
	Red blood cell	104,57€	[19,21]
Imaging(per exam.)	Chest radiograph	22,49€	[19,20]
	Colonoscopy	121,58€	[19,20]
	Computed Tomography	83,20€	[19,20]
	Ultrasound	50,48€	[19,20]
	Magnetic Resonance Imaging	139,50€	[19,20]

Relapse-free survival three years after diagnosis was taken as the indicator of effectiveness. Mean relapse-free survival was calculated for CPG-compliant and non compliant groups.

### Statistical analysis

Chi-square or t-tests were used, according to the type of data, to compare compliant (CPG+) and non compliant (CPG-) groups. Analysis of variance (ANOVA) was used to explore the costs of overall management according to histological subtype. Univariate survival analyses were performed using the Kaplan-Meier method and the log-rank test. The log-rank test was used to assess the effect of compliance with CPGs on survival after adjusting for confounding variables.

The incremental cost-effectiveness ratio (ICER), expressing the incremental cost per additional relapse-free year gained.

**Figure 1:**
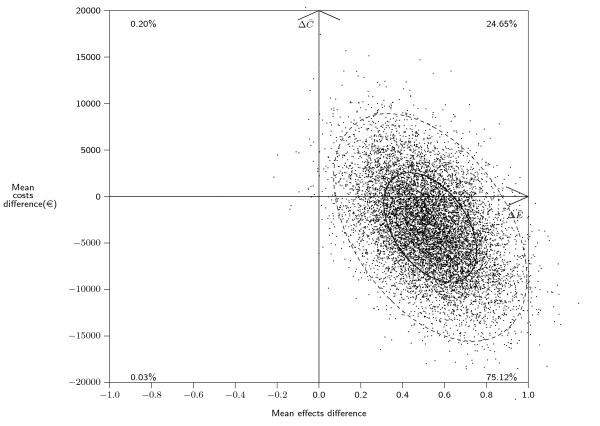
**Probabilistic analysis of the ICER: scatter of points and confidence ellipses**.

The uncertainty surrounding the ICER was captured by a probabilistic sensitivity analysis according to procedures established by the French National Authority for Health (HAS) [22]. Ten thousand replications were obtained by the non parametric bootstrap method. A graphical representation of the sampling uncertainty associated with the ICER on the cost-effectiveness plane is shown in Figure 1[23]. The four quadrants of the cost-effectiveness plane are as follows: northeast, compliance with CPGs more costly and more effective than non compliance; southeast, compliance less costly, more effective; northwest (more costly, less effective) and southwest (less costly, less effective). Confidence regions were assessed and are represented by ellipses.

The outer ellipse defines the confidence region for the mean cost difference and mean effect difference pair at the 95% level and the inner ellipse at the 50% level. The outer ellipse is equivalent to an acceptability region for an inference test at 5% significance level whose null hypothesis is the mean cost difference and mean effect difference pair being equal to (0, 0). Consequently, if the origin of the cost-effectiveness plane does not belong to the confidence ellipse, then both the management of patients with sarcoma (adherence versus non adherence to CPGs) are significantly different regarding costs and effects. For more robustness in the results, in addition to confidence ellipses, uncertainty around the ICER was taken into account by examining probabilities that it belonged to each of the quadrants of the cost-effectiveness plane.

Calculations were performed using STATA 11 and Gauss software version 9.0.

## Results

Table 3 shows the key characteristics of patients and the health outcome in the whole of the study population (219 cases) and in the CPG + (n = 118) and CPG- (n = 101) groups. Overall, age at diagnosis, ranging from 18 to 94 years, was in average 60 years and the majority of patients studied (55%) were women. There was a variety of histological types, the most frequent being Gastro Intestinal Stromal Tumors(GIST) (26%), liposarcomas (21%) and leiomyosarcomas (12%). Sixtythree percent of tumors were of soft tissue, mostly in the trunk, and 81% deep seated. Tumors had a mean size of 95 mm (range 3 to 450 mm). Tumors were grade II/III in 58% of cases, Thirtytwo patients (15%) had previous cancers. Differences between CPG compliant and non compliant groups were significant for grade (there were more high grade tumors in patients whose management did not comply with CPGs, *p *= 0.01) and histological type (*p *= 0.03): CPGs was less likely in liposarcoma and leiomyosarcoma. Relapse free survival was a mean of 2.46 years in the CPG + group and 2.16 years in the non-compliant group (*p *= 0.04).

**Table 3:** Clinical characteristics of patients by group: Mean ± SD or number of patients and (%)

Clinical characteristics		All patients n = 219	CPG (+) n = 118	CPG (-) n = 101	p
Age (years)		60.4 ± 15.0	60.1 ± 15.3	60.7 ± 14.8	0.39
Sex (females)		121 (55.2%)	66 (56.0%)	55 (54.5%)	0.83
Previous cancer		32 (14.8%)	15 (12.8%)	17 (17.2%)	0.37
Tumour grade	low (grade I)	38 (20.3%)	27 (27.6%)	11 (12.4%)	0.01
	intermediate (grade II)	38 (20.3%)	18 (18.3%)	20 (22.5%)	
	high (grade III)	70 (37.5%)	27 (27.6%)	43 (48.3%)	
	not applicable	41 (21.9%)	26 (26.5%)	15 (16.8%)	
Tumour site	soft tissues	137 (62.6%)	71 (60.2%)	66 (65.4%)	0.43
	viscera	82 (37.4%)	47 (39.8%)	35 (34.6%)	
Tumour size in mm		94.6 ± 80.6	96.8 ± 91.4	91.9 ± 66.3	0.67
Tumour depth	superficial	38 (18.9%)	22 (20.6%)	16 (17%)	0.52
depth	deep	163 (81.1%)	85 (79.4%)	78 (83%)	
Histology	GIST	54 (25.6%)	37 (32.5%)	17 (17.5%)	0.03
	Liposarcoma	45 (21.3%)	18 (15.8%)	27 (27.8%)	
	Leiomyosarcoma	25 (11.8%)	9 (7.9%)	16 (16.5%)	
	Sarcoma NOS	16 (7.6%)	9 (7.9%)	7 (7.2%)	
	Dermatofibrosarcoma	16 (7.6%)	11 (9.6%)	5 (5.2%)	
	Others	55 (26.1%)	30 (26.3%)	25 (25.8%)	
Relapse free survival (years)		2.40	2.46	2.16	0.04

NOS: Not Otherwise Specified

GIST: Gastro Intestinal Stromal Tumours

Table 4 shows details of the mean costs in CPG + and CPG- groups. The overall (for all 219 cases) cost of sarcoma management per patient, ranging from €1,303 to €107,464, reached €25,296 in average. Surgery (primary and wide surgical excisions), with an average cost of €8,170 represented 32% of the average total cost. Chemotherapy (€6,107) accounted for 24% of the average total cost, follow up (€5,048) for 20% of the average total cost and diagnosis (€3,701) for 15%. Radiotherapy (€2,270) represented 9% of the average total cost. Overall average cost per patient of managing sarcomas (all subtypes included) was somewhat less in the GPG + group than in the CPG- group, but the difference did not reach statistical significance (*p *= 0.07). Overall average cost was significantly lower in the GPG + group than in the CPG- group for GIST and dermatofibrosarcoma (*p *< 0.01).

**Table 4:** Average costs for each phase of sarcoma management by group (in €, 2009)

Phases of treatment		All patients	CPG (+)	CPG (-)	p
Diagnosis	overall	3,701 (7420)	4,536 (8,908)	2,728 (5,095)	0.07
	hospitalization	3,275 (7,376)	4,097 (8,791)	2,317 (4,701)	0.07
	imaging	259 (198)	274 (210)	241 (182)	0.21
	biopsy	93 (57)	88 (45)	99 (69)	0.15
	consultation	74 (96)	77 (88)	71 (105)	0.64
Surgery	overall	8,170 (7,364)	7,397 (6,034)	9,075 (8,639)	0.09
	hospitalization	7,927 (7,101)	7,163 (5,773)	8,821 (8,363)	0.09
	imaging	95 (163)	108 (179)	80 (141)	0.21
	transfusion	148 (384)	126 (384)	174 (386)	0.36
Chemo-therapy	overall	6,107 (11,988)	5,164 (11,197)	7,207 (13,123)	0.22
	hospitalization	3,913 (8,568)	2,938 (7,176)	5,050 (9,905)	0.07
	consultation	59 (405)	81 (545)	35 (98)	0.39
	transfusion	56 (223)	53 (228)	60 (220)	0.20
	drugs	1,956 (5,171)	1,982 (5,964)	1,925 (4,115)	0.94
	imaging	123 (279)	110 (277)	137 (284)	0.48
Radio-therapy	overall	2,270 (5,850)	2,078 (5,599)	2,493 (6,173)	0.60
	hospitalization	1,544 (5,223)	1,449 (4,867)	1,656 (5,658)	0.77
	sessions	726 (1,266)	629 (1,269)	837 (1,262)	0.23
Initial treatment		20,248 (18,474)	19,175 (17,555)	21,502 (19,778)	0.36
Follow Up		5,048 (11,760)	4,396 (12,343)	5,811 (11,116)	0.38
Overall management		25,296 (22,919)	23,571 (21,913)	27,313 (24,403)	0.23

Table 5 shows that compliance with CPGs was less costly and more effective than non compliance. On the basis of the ICER point estimate, clinicians' adherence to CPGs in the management of patients with sarcoma strictly dominates non adherence for relapse free survival. Since the origin of the cost-effectiveness plane presented in Figure 1 was not included in the inner 95% confidence ellipse, clinicians' adherence versus non adherence to CPGs is significantly different in terms of cost and effectiveness. The probability of the ICER belonging to each quadrant of the cost-effectiveness plane is highest (75%) for the southeast quadrant, in which compliance with CPG is both less costly and more effective than non compliance. This is in accordance with the results of Table 5 also concluding for less costly and more effectiveness in compliant group.

**Table 5:** Incremental cost-effectiveness ratio (ICER) for clinicians' adherence versus non adherence to clinical practice guidelines (CPGs)

	Mean cost per patient(€, 2009)	Mean Incremental Cost[[]](€, 2009)	Mean effectiveness per patient(Relapse-free survival, years)	Mean Incremental Effectiveness[[]](Relapse-free survival, years)	ICER(€ per relapse-free year gained)[[]]
Overall management CPG (+)	23,571	-	2.46	-	
Overall management CPG (-)	27,313	3,742	2.16	-0.30	Dominated

## Discussion

A weakness of the present study is that its retrospective nature did not allow assessment of outcome in cost per Quality Adjusted Life Year (QALY), as recommended by the National Institute for Health and Clinical Excellence (NICE) [24,25]. Notwithstanding this, freedom from relapse undoubtedly contributes to quality of life in cancer patients.

Another weakness was the lack of information on potentially confounding variables such as the initial performance status of the patient, the cumulative volume for the surgeon and the hospital, the type of provider (academic cancer centre, non academic cancer centre, non academic non cancer centre) [26-28] that could not be controlled for in the statistical analysis. Any resulting bias, however, would have tended to cause an underestimation rather than an overestimation of the true effect of compliance, since in the CPG + group the costs of diagnosis were higher (Table 4) despite a lower grade of tumours (Table 3).

Interestingly, the higher overall cost involved in diagnosing patients according to CPGs was accounted for almost entirely by increased hospitalisation (rather than by greater use of imaging or biopsy or external consultations) (Table 4). The average cost of hospitalization was €4,097 in CPG + and €2,317 in CPG- groups. The difference corresponds to 2.3 additional days of hospital stay at the prevailing daily cost of €760 per day (Table 2). Adherence to CPGs requires that decisions be made within a multidisciplinary committee, and it is likely that patients' hospitalization was prolonged in order to schedule the multidisciplinary meeting needed to reach a consensus on treatment.

A greater adherence to CPGs during diagnosis appears to decrease all subsequent costs, notably those of surgery and chemotherapy, probably by reducing the need for surgical re-intervention or more intense use of antineoplastic drugs (Table 4). Surgery and chemotherapy were the main factors driving the cost of initial treatment, representing about 66% of the overall cost in the CPG + group ((7,397 + 5,164)/19,175) and 76% ((9,075 + 7,207)/21,502) in the CPG- group.

In contrast to other studies that have shown it may be cheaper in the short term to deviate from CPGs [29], the present study found that compliance with CPGs strictly dominates for relapse-free survival, meaning that management of sarcoma according to CPGs is less costly and more effective. This evidence should encourage health providers to promote adherence to guidelines since adopting this approach achieves a better quality of care and is a more efficient allocation of resources. It should also encourage compliance among physicians, some of whom perceive CPGs as limiting their freedom to make diagnostic and therapeutic decisions.

Compliance with CPGs in sarcoma management has increased since 2001. A retrospective study of the medical records of sarcoma patients in the University Hospital of Lyon and the Cancer Centre Léon Bérard found that between 1999 and 2001 initial clinical management had been consistent with the CPG in only 32% of cases [12]. In this study, the compliance rate reached 54%. Compliance with guidelines was greater for chemotherapy than for other aspects of sarcoma management since detailed protocols are more common than in diagnosis, surgery, radiotherapy or follow up. Compliance with CPGs was also more frequent for low-grade than for high-grade sarcomas (*p *= 0.01); and it has been suggested that physicians are less likely to adhere to CPGs if they believe that compliance will not improve outcome [30]. Compliance was also better for GIST than for other histologies, probably because imatinib and related drugs have led to a remarkable improvement in management on which there is global consensus [31].

## Conclusions

The present findings should encourage physicians' efforts to increase their compliance with CPGs and encourage healthcare administrators to invest in implementing CPGs in the management of sarcoma.

## Abbreviations

ANOVA: Analysis of variance; CEA: Cost-effectiveness analysis; CPG: Clinical practice guidelines; CPG +: Clinicians' adherent to practice guidelines; CPG-: Clinicians' non adherent to practice guidelines; CONTICANET: Connective tissues cancers network; GIST: Gastro Intestinal Stromal Tumours; HAS: French National Authority for Health (Haute Autorité de Santé); ICER: Incremental cost-effectiveness ratio; NICE: National Institute for Health and Clinical Excellence; NOS: Not otherwise specified; QALY: Quality adjusted life year

## Competing interests

The authors declare that they have no competing interests.

## Authors' contributions

LP, AB, GM designed the study, acquired and interpreted the clinical and cost data, undertook the statistical analysis, and prepared the manuscript. AV, PS, FD participated in clinical data acquisition and analysis. FNG carried out surgical data acquisition and analysis, PB chemotherapy data acquisition and analysis, DRV, AVD, APDT, JMC, and PT diagnosis data acquisition and analysis, and CB the analysis of data from young patients. CS participated in the statistical analysis. JYB, CRR and IRC participated in general CONTICANET coordination as well as study design and compliance supervision. All authors read and approved the final manuscript.

## Pre-publication history

The pre-publication history for this paper can be accessed here:

http://www.biomedcentral.com/1472-6963/12/82/prepub

## Appendix A. Annexe 1. Main CPG criteria for each sequence of initial sarcoma treatment

Main Criteria for diagnosis: Clinical size and depth of the tumour mass must be recorded; Computed Tomography (CT) is required for abdominal localizations, or Magnetic Resonance Imaging (MRI) for limb localizations; Chest radiograph or CT scan is required to identify metastases; Initial biopsy (incisional or needle), preferably by the surgeon in charge of future surgical procedures, is required for bone and soft tissue sarcomas, with the exception of small tumours (< 3 cm) for which excisional biopsy is considered appropriate.

Main Criteria for surgery: Whenever possible, primary surgery should involve a wide excision with 1-2 cm margins. For high-grade, large (> 3 cm) or deep-seated tumours, surgery alone is acceptable only in case of amputation or compartmental resection with negative histological margins (R0). Wide excision alone, with no adjuvant treatment, is acceptable only for superficial, small (< 3 cm) and low-grade lesions. Histologically positive margins (R1) or incomplete excision (R2) have to be considered inadequate, and should be followed by further appropriate treatment.

Main criteria for chemotherapy: For non-readily operable sarcomas, primary chemotherapy or radiation therapy can be an option. For readily operable sarcomas, neo-adjuvant chemotherapy should be performed only as part of a clinical research protocol. In the adjuvant setting, systemic chemotherapy should be performed only within the context of a prospective clinical trial. Adjuvant chemotherapy can be performed for patients with histologically positive margins after wide surgical excision.

Main criteria for radiation therapy: Association of wide surgical excision and adjuvant radiation therapy should be considered the standard treatment. The absence of adjuvant radiotherapy is acceptable for superficial, small (< 3 cm) and low-grade tumours, and for limb sarcomas when amputation is performed. For non-operable sarcomas, primary radiation therapy could be an option. The optimal treatment strategy involves a 50 Gy delivered dose with an additional boost of 10 Gy in case of microscopic residual tumour (R1), with a target volume encompassing the tumour bed and surgical scars, including draining orifices, with adapted security margins. Moreover, the interval from surgery to radiation therapy must not be longer than 8 weeks.
